# Multiplex one-step RT–qPCR assays for simultaneous detection of BRV, BCoV, *Escherichia coli* K99^+^ and *Cryptosporidium parvum*

**DOI:** 10.3389/fvets.2025.1561533

**Published:** 2025-03-26

**Authors:** Xinru Zhao, Min Li, Yingying Yang, Yidan Wang, Xiaoru Zheng, Dehua Yin, Haihui Gao, Huatao Li, Kaiqiang Fu, Zhi Cao

**Affiliations:** ^1^College of Veterinary Medicine, Qingdao Agricultural University, Qingdao, China; ^2^Innovus Solarex Biotech Co., Ltd., Qingdao, China; ^3^Ningxia Academy of Agricultural and Forestry Sciences, Ningxia, China

**Keywords:** BRV, BCoV, *E. coli* K99^+^, *C. parvum*, multiplex *Taq*Man real-time PCR

## Abstract

**Introduction:**

Bovine rotavirus (BRV), bovine coronavirus (BCoV), *Escherichia coli* K99^+^ (*E. coli* K99^+^), and *Cryptosporidium parvum* (*C. parvum*) are the most common pathogens involved in calf production. These pathogens can cause calf diarrhea, leading to significant economic losses in the cattle farming industry. These four pathogens have similar clinical symptoms, making them difficult to distinguish. Therefore, we established a one-step quadruple *Taq*Man fluorescence quantitative PCR method capable of simultaneously and rapidly detecting BRV, BCoV, *E. coli* K99^+^, and *C. parvum*.

**Methods:**

Specific primers and *Taq*Man probes were designed for the BRV VP-6 gene, BCoV N gene, *E. coli* K99^+^ K99 gene, and *C. parvum* 18S rRNA gene. Standard positive plasmids were constructed, and the reaction conditions of the method were optimized. The sensitivity, specificity, and repeatability of the method were validated, and clinical samples were tested.

**Results:**

The minimum detection limits of this method for BRV, BCoV, *E. coli* K99^+^, and *C. parvum* were 5.8 × 10^1^, 2.3 × 10^1^, 4.5 × 10^2^, and 2.6 × 10^1^ copies/μL, respectively. The intra- and intergroup coefficients of variation were all less than 1.2%. This method has the advantages of strong specificity, reproducibility, low cost, and no cross-reaction with other bovine pathogens. Compared with the commercial reagent kit method were used to analyze clinical samples, and both the diagnostic sensitivity (DSe) and diagnostic specificity (DSp) were above 90%, with kappa values greater than 0.9.

**Discussion:**

The one-step multiplex RT-qPCR method developed in this study for detecting BRV, BCoV, *E. coli* K99^+^, and *C. parvum* is expected to be an effective tool for the rapid and economical diagnosis and monitoring of diarrhoeal diseases in calves.

## 1 Introduction

Neonatal calf diarrhea (NCD) is one of the most serious diseases of calves worldwide due to high mortality and stunted growth of affected animals, resulting in productivity and economic losses for cattle producers worldwide (1–3). The causes of bovine diarrhea include both infectious and noninfectious factors. Infectious factors include bacteria, viruses, parasites and other pathogens. Bovine rotavirus, Bovine coronavirus, *E. coli* K99^+^ and *Cryptosporidium* are the most common pathogenic factors of calf diarrhea. BRV is frequently involved in mixed infections (4, 5).

Bovine rotavirus, a member of the genus *Rotavirus* within the *Reoviridae* family and is the main cause of calf diarrhea. It causes symptoms such as diarrhea, loss of appetite, dehydration and depression. Often leading to secondary bacterial infection due to compromised immune resistance. In addition, there are potential zoonotic and economic impacts (6–8).

Bovine coronavirus, belonging to the genus *Coronavirus* within the *Coronaviridae* family, is an enveloped, linear single-stranded RNA virus. Most calves develop yellow watery diarrhea accompanied by mucus and blood clots, dehydration, hypothermia, depression, reduced food intake and electrolyte disturbance, which can lead to metabolic acidosis and hypoglycaemia (9, 10).

Bovine colibacillosis, an economically significant disease in newborn calves, is caused by enterotoxigenic *Escherichia coli* (ETEC). The most common strain of ETEC expresses F5 (K99) fimbriae (11), which allows the bacteria to attach to intestinal cells. It can cause severe diarrhea and rapid dehydration of newborn calves, and hinder the growth and development of calves (12).

The main *Cryptosporidium* species infecting cattle include *C. parvum*, *C. bovis*, *C. andersoni*, and *C. ryanae*. *C. parvum* mainly infects young calves, and it is the most important zoonotic disease (13–15). Clinical symptoms include malabsorption and secretory diarrhea, which usually resolve on their own within 2 weeks. Diarrhea severity can range from mild to severe, with yellowish watery or mucoid feces. Affected calves may exhibit dehydration, depression, and anorexia, depressed, and anorexic (16).

Studies on calf diarrhea in Ningxia (2021–2022) found detection rates of 50.46% for *Cryptosporidium*, 23.18% for BRV, 20% for *E. coli* K99, and 11.82% for BCoV (17); A systematic review and meta-analysis estimated the prevalence of BRV in 25 provinces of China from 1984 to 2021, with a prevalence rate of 35.7% (8,176/17,292) (18). Another study collected 1,646 cow fecal samples, with a detection rate of 34.02% for BCoV (19). A study investigated diarrheal samples from calves treated at the University of Etchiers, screening for BCoV, *Cryptosporidium*, *E. coli* K99^+^, and BRV. The results showed that *Cryptosporidium* was the most common pathogen (61.5%), followed by rotavirus (56.4%) (20). A global meta-analysis (2000–2021) on bovine *C. parvum* in newborn calves reported that 33.6% (1,637/6,077) of diarrheic calves were infected (21).

These pathogens cause bovine diarrhea in clinical practice, and the symptoms are similar; moreover, they are difficult to distinguish, and there are many mixed infections, so it is necessary to carry out pathogen detection for differential diagnosis to improve targeted prevention and treatment. The common detection methods for these four pathogens include PCR, ELISA and LAMP, etc. (22–27). PCR detection may require a longer time to obtain results, with complex operating steps and the need to run agarose gel electrophoresis to visualize the results. ELISA has relatively lower detection precision and sensitivity compared to molecular biology methods, and involves more experimental steps. LAMP has several disadvantages, such as complex primer design, complicated product structure, difficulty in quantification, and higher risk of false positives. In comparison, real-time quantitative PCR (qPCR) is a well-established method for the detection, quantification, and typing of different microbial agents in the areas of clinical and veterinary diagnostics and food safety (28). At present, different methods have been established to detect the above four pathogens (29–32); however, there is no rapid simultaneous detection method to distinguish among the four pathogens at the same time. Therefore, the aim of this study was to establish a one-step multiplex *Taq*Man fluorescent quantitative PCR method for BCoV, BRV, *E. coli* K99^+^, and *C. parvum* to make the laboratory diagnosis of bovine more convenient and rapid and minimize the impact of bovine diarrhoeal diseases.

## 2 Materials and methods

### 2.1 Primer design and synthesis

The VP6 gene of representative BRV strains (JN790188.1, AF317127.1, EU873011.1, etc.), the N gene of BCoV strains (EF424617.1, OP037373.1, LC494170.1, etc.), the K99 gene of *E. coli* K99*^+^* strains (JX987524.1, MF467447.1, MF467448.1, etc.), and the 18S rRNA gene of *C. parvum* strains (MZ892386.1, AB513858.1, MZ377024.1, etc.) were analyzed using Primer Express 5.0 software to identify conserved regions (Table 1). Primers and probes were designed based on these conserved sequences and synthesized by Sangon Biotech (Shanghai) Co., Ltd.

**Table 1:** Multiple *Taq*Man fluorescence quantitative PCR primer and probe sequences.

Pathogen	Target gene	Sequence (5′ to 3′)	Reporter/quencher
BRV	VP6	F ATTGTCGAAGGCACATTATACTCCA	
		R ATTGCGAGCCGTTTCAACATAG	
		P GGGGGAATAGGTAATCTACCGATTA	FAM/BHQ1
BCoV	N	F GCCACAGCAAGTAACTAAGCAG	
		R TTTAACATTTCTCCACCACCAA	
		P GTTCAGCAGTGTTTTGGTAAGAGAG	VIC/BHQ1
*E. coli* K99^+^	K99	F AGGTCAATGGTAATCGTACATCAAC	
		R GGTATCCTTTAGCAGCAGTATTTC	
		P AAAACAAATGCTCGTATTGACTGG	ROX/BHQ2
*C. parvum*	18S rRNA	F GAAAGCATTTGCCAAGGATGT	
		R GTCTGGACCTGGTGAGTT	
		P TCAGCCTTGCGACCATACTCC	CY5/BHQ2

### 2.2 Sample source, pathogen strains and nucleic acid

Four hundred clinical diarrhea samples were obtained from intensive farms in Shandong Province. The nucleic acid samples of BRV, BCoV, *E. coli* K99^+^, *C. parvum*, Bovine Parvovirus (BPV), Infectious Bovine Rhinotracheitis Virus (IBRV), and Bovine Nebovirus (BNeV) were obtained from field isolates and preserved at the College of Veterinary Medicine, Qingdao Agricultural University (QAU). In addition, the samples of *Salmonella typhimurium* (*S. typhimurium*) (ATCC13311) and *Pasteurella multocida* (*P. multocida*) (ATCC12948) were obtained from the College of Veterinary Medicine, Qingdao Agricultural University (QAU).

### 2.3 Extraction of pathogenic nucleic acid template

The collected feces were placed in physiological saline, and pathogenic DNA and RNA were manually extracted from clinical samples according to the instructions of the VAMNE Magnetic Pathogen DNA/RNA Kit (Vazyme, China). The extracted DNA/RNA samples were used as templates for qPCR detection.

### 2.4 Preparation of standard positive plasmids

Genomic RNA from BRV and BCoV was extracted and reverse-transcribed into cDNA using the FastKing RT Kit (TIANGEN Biotech, China). Genomic DNA from *E. coli* K99^+^ and *C. parvum* was also extracted. Specific primers were used to amplify the target genes using cDNA/DNA as templates, the target products were purified, the target genes were transferred into DH5α competent cells (Thermo Fisher, China) for enhanced culture, and the cells were cultured in a rocking incubator at 37°C overnight. Positive clones were selected through blue-white screening and identified as positive by PCR. Subsequently, the samples were sent to Sangon Biotech Co., Ltd. in Shanghai for sequencing. Use the nanodrop 2000 spectrophotometer (Thermo Fisher, China) to measure the concentration and calculate the copy number.

### 2.5 Reaction condition optimization

Using BRV, BCoV, *E. coli* K99^+^, and *C. parvum* positive plasmids as templates, 5 μL of each template was added to different reaction systems to screen for optimal primer and probe concentrations. The amplification conditions were as follows: 37°C for 2 min; 52°C for 5 min; 95°C for 1 min; 95°C for 3 s; and 60°C for 30 s. Using an orthogonal design for optimization, qPCR amplification was performed for 40 cycles with the TOROIVD® Probe 1-step RT-qPCR 5G Kit 3.0 (TOROIVD, China) on a Gentier 96R instrument (TIANLONG, China) to determine the optimal concentrations of primers and probes in the reaction system.

### 2.6 Standard curve establishment

The four recombinant standard plasmids were serially diluted to 10^8^–10^2^copies/μL in a 10-fold gradient and used as templates for qPCR amplification, with ddH_2_O as a negative control. Amplification was performed using the CFX Opus Real-Time PCR System (BIO-RAD, China). A standard curve was generated by plotting the template concentration on the x-axis and the corresponding Ct values on the y-axis to evaluate the linear range and amplification efficiency of the assay.

### 2.7 Specificity assay

The specificity of the optimized *Taq*Man qPCR method was validated using *E. coli* K99^+^, *C. parvum*, BPV, *S. typhimurium*, and *P. multocida* genomic DNA, BRV, BCoV, IBRV, and BNeV cDNA as templates, with ddH_2_O as a negative control.

### 2.8 Sensitivity assay

The prepared positive plasmids were serially diluted in a 10-fold manner. Singleplex RT-qPCR amplifications were performed using positive plasmids with concentrations ranging from 10^4^ to 10^1^ copies/μL as templates, with ddH₂O serving as the negative control. Additionally, multiplex RT-qPCR amplifications were conducted using a mixture of positive plasmids for BRV, BCoV, *E. coli* K99^+^, and *C. parvum*, with copy numbers ranging from 10^4^ to 10^1^ as templates, and ddH₂O as the negative control.

### 2.9 Reproducibility of the *Taq*Man qPCR assay

The four recombinant plasmids were diluted, and three different concentrations 10^4^–10^2^ copies/μL were selected as positive templates, with nuclease-free water as the negative control. Multiplex fluorescent quantitative PCR amplification was performed within the same batch and across different batches. The coefficient of variation calculation formula CV = (standard deviation s/mean x) × 100% was used to calculate the coefficient of variation in the intrabatch and interbatch repeatability tests, and the repeatability of the method was evaluated.

### 2.10 Validation of diagnostic specificity and sensitivity

A total of 400 samples of calf diarrhea collected from large-scale cattle farms in Shandong Province from December 2023 to March 2024 were detected using the proposed method to investigate the prevalence of the four pathogens in this area and the results were compared with TOROIVD® Gene Test Tube (TOROIVD®, China). DSe and DSp evaluations were conducted via the proposed method. Kappa was further used to test the consistency of the two detection methods.

### 2.11 Statistical analysis

The formula for calculating diagnostic specificity (DSe) and diagnostic sensitivity (DSp) is DSe = TP/(TP + FN) and DSp = TN/(TN + FP), where a represents a true positive, c represents a false-negative, b represents a false-positive, and d represents a true negative. Kappa = [Pr(a)-Pr(e)]/[1-Pr(e)], where Pr(a) represents the actual observed consistency rate and Pr(e) represents the chance consistency rate.

## 3 Results

### 3.1 Construction of standard positive plasmids

After the recombinant plasmid was constructed, amplified, and purified, BLAST comparison was carried out, and if the results were consistent with the target sequence, the recombinant plasmid was successfully constructed. The concentration of the recombinant plasmid was calculated, and the concentrations of BRV, BCoV, *E. coli* K99^+^ and *C. parvum* were adjusted to the following: 5.8 × 10^10^, 2.3 × 10^10^, 4.5 × 10^10^ and 2.6 × 10^10^ copies/μL, respectively.

### 3.2 Optimization of the reaction conditions

The optimal primers (50 μmol/L) for BRV, BCoV, *E. coli* K99^+^ and *C. parvum* were 0.18 μL, 0.22 μL, 0.18 μL and 0.20 μL, and the optimal probe combinations were 0.6 μL, 0.8 μL, 0.6 μL and 0.4 μL. The reaction system consisted of 12.5 μL of 2 × 5G qPCR Buffer BB, 1.3 μL of RT–qPCR Enzyme Mix, 5 μL of template and ddH_2_O to bring the final volume to 25 μL.

### 3.3 Establishment of a standard curve

**Figure 1:**
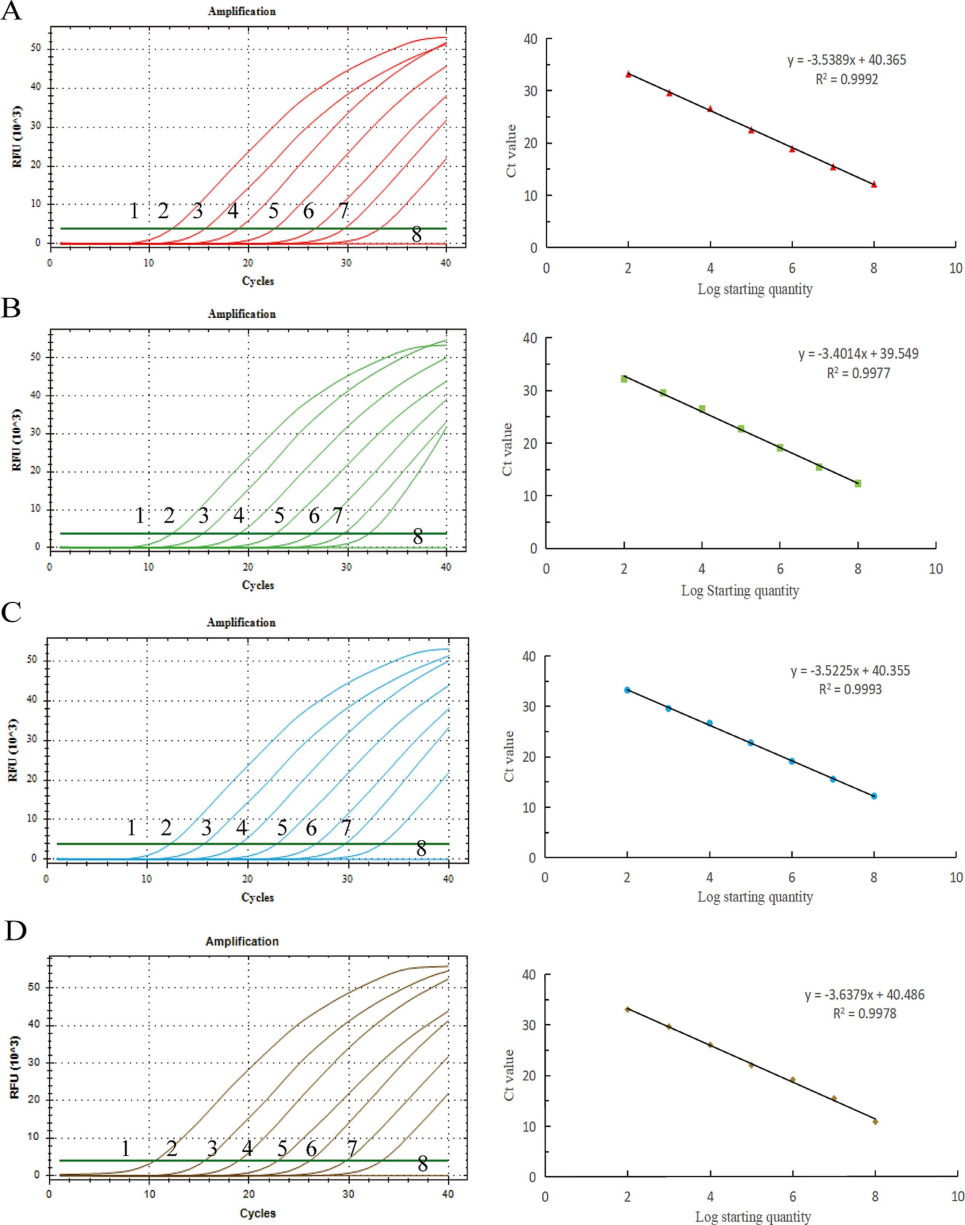
Standard curves of the developed *Taq*Man real-time PCR system for detection. X-axis: copy number; Y-axis: cycle number. **(A)** BRV, **(B)** BCoV, **(C)** *E. coli* K99^+^, **(D)** *C. parvum*. y: the curve from the equation, *R*^2^: correlation coefficient.

Copy numbers 10^8^–10^2^ copies/μL were selected as the reaction template for qPCR amplification, and the standard curve was established with positive plasmid copy numbers as the horizontal coordinate and corresponding Ct values as the vertical coordinate (Figure 1). The BRV linear regression equation was *y* = −3.5389x + 40.365, and the correlation coefficient *R*^2^ = 0.9992. The linear regression equation of BCoV was *y* = −3.4014x + 39.549, and the correlation coefficient *R*^2^ = 0.9977. The linear regression equation of *E. coli* K99^+^ was *y* = −3.5225x + 40.355, and the correlation coefficient *R*^2^ = 0.9993. The linear regression equation of *C. parvum* was *y* = −3.6379x + 40.486, and the correlation coefficient *R*^2^ = 0.9978.

### 3.4 Specificity analysis

**Figure 2:**
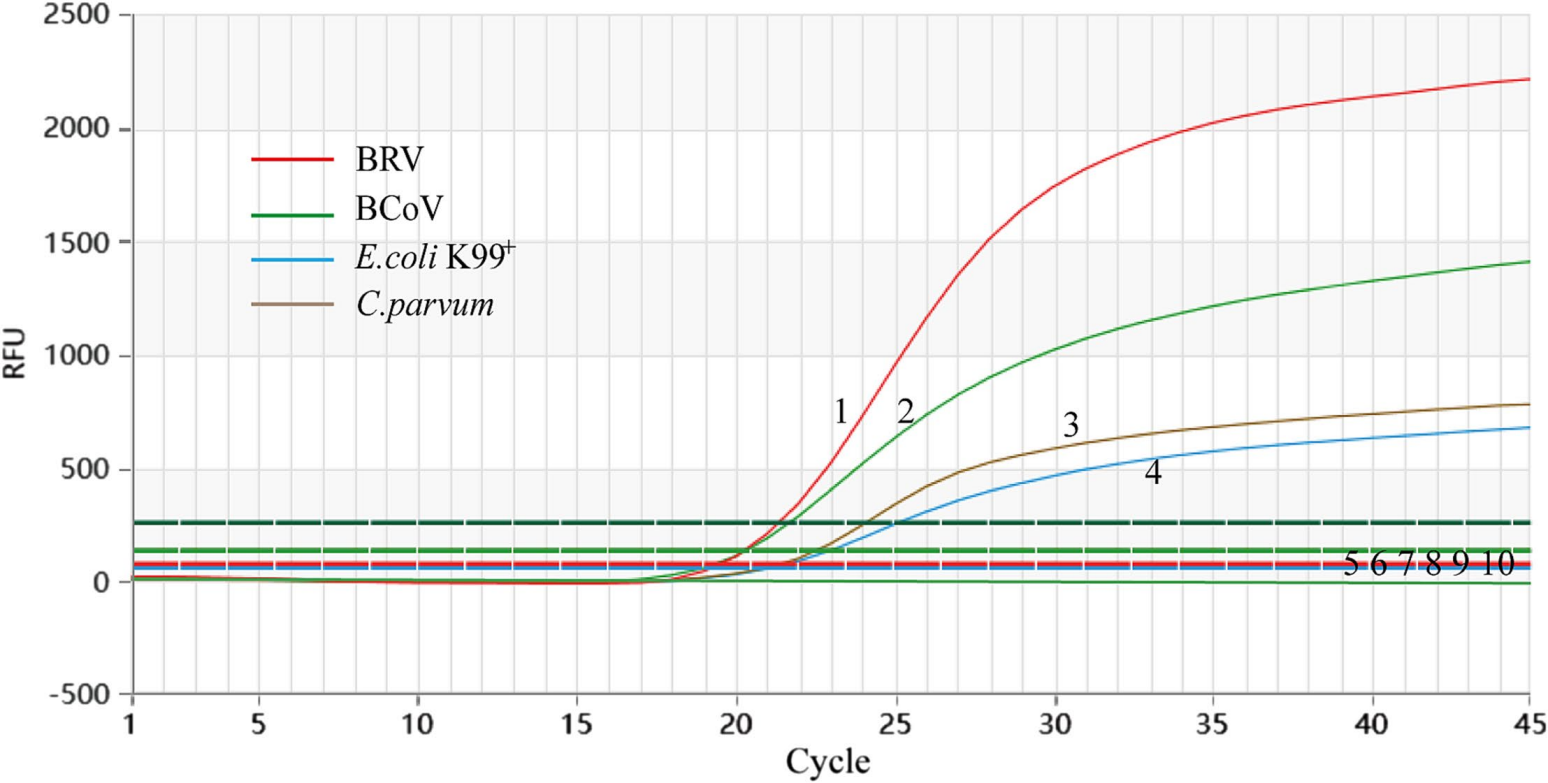
Analytical specificity of the primer/probe sets used in the one-step real-time RT-PCR assay. 1–4: BRV, BCoV, *C. parvum,* and *E. coli* K99^+^. 5–10: *S. typhimurium*, *P. multocida*, IBRV, BNeV, BPV, ddH_2_O.

BRV, BCoV, *E. coli* K99^+^, and *C. parvum* all presented Ct values and corresponding amplification curves, whereas no amplification curves were found for the negative reference products, indicating that the experiment was successful (Figure 2).

### 3.5 Sensitivity analysis

**Figure 3:**
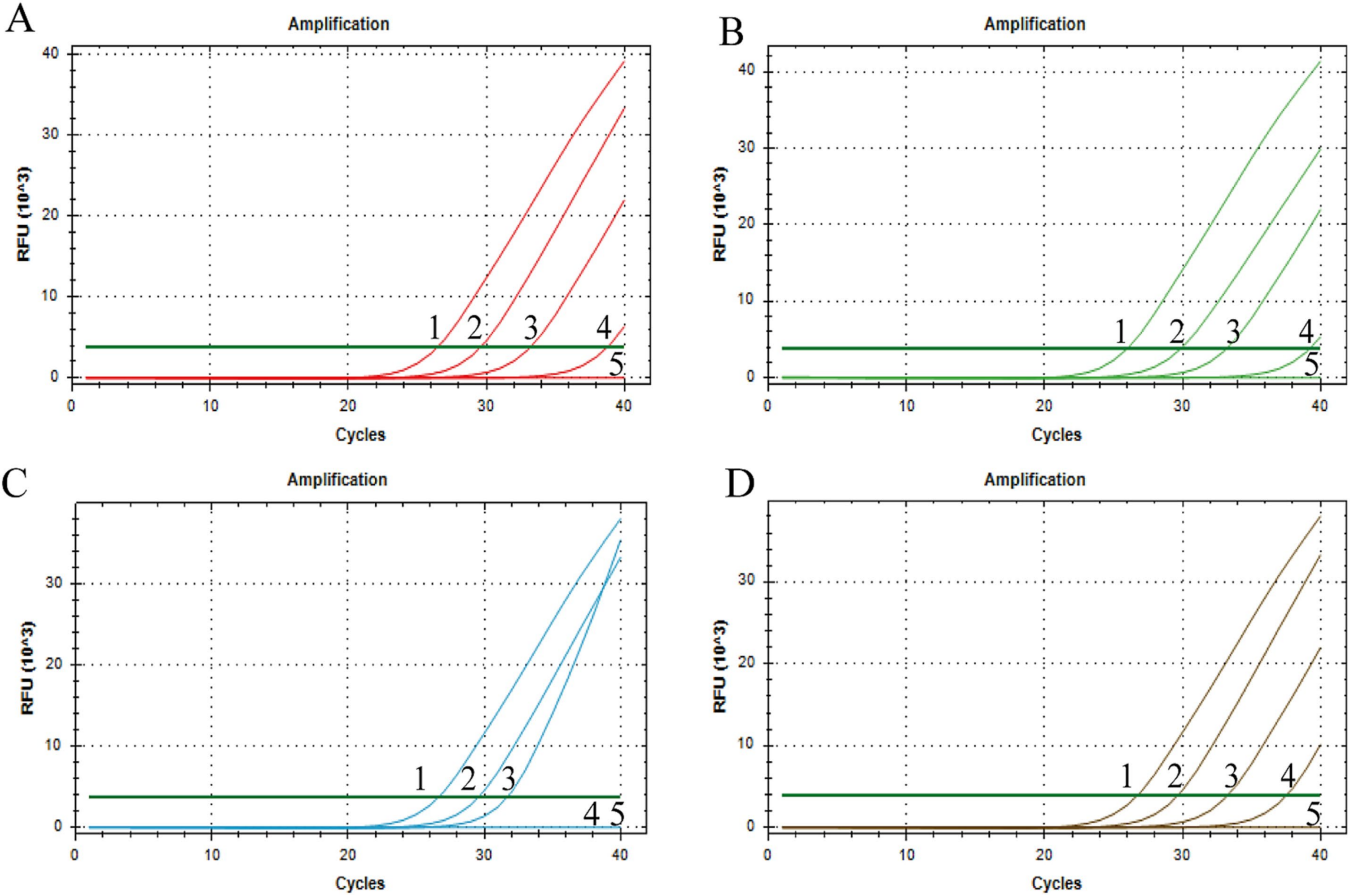
Sensitivity test of singleplex TaqMan Real-Time PCR Assay. X-axis: number of cycles; Y-axis: RFU; **(A)** BRV, **(B)** BCoV, **(C)** *E. coli* K99^+^, **(D)** C. *parvum*; 1–4: fluorescence quantitative PCR amplification curve of each standard positive recombinant plasmid from 10^4^ to 10^1^ copies/μL of plasmid content; 5: ddH_2_O.

**Figure 4:**
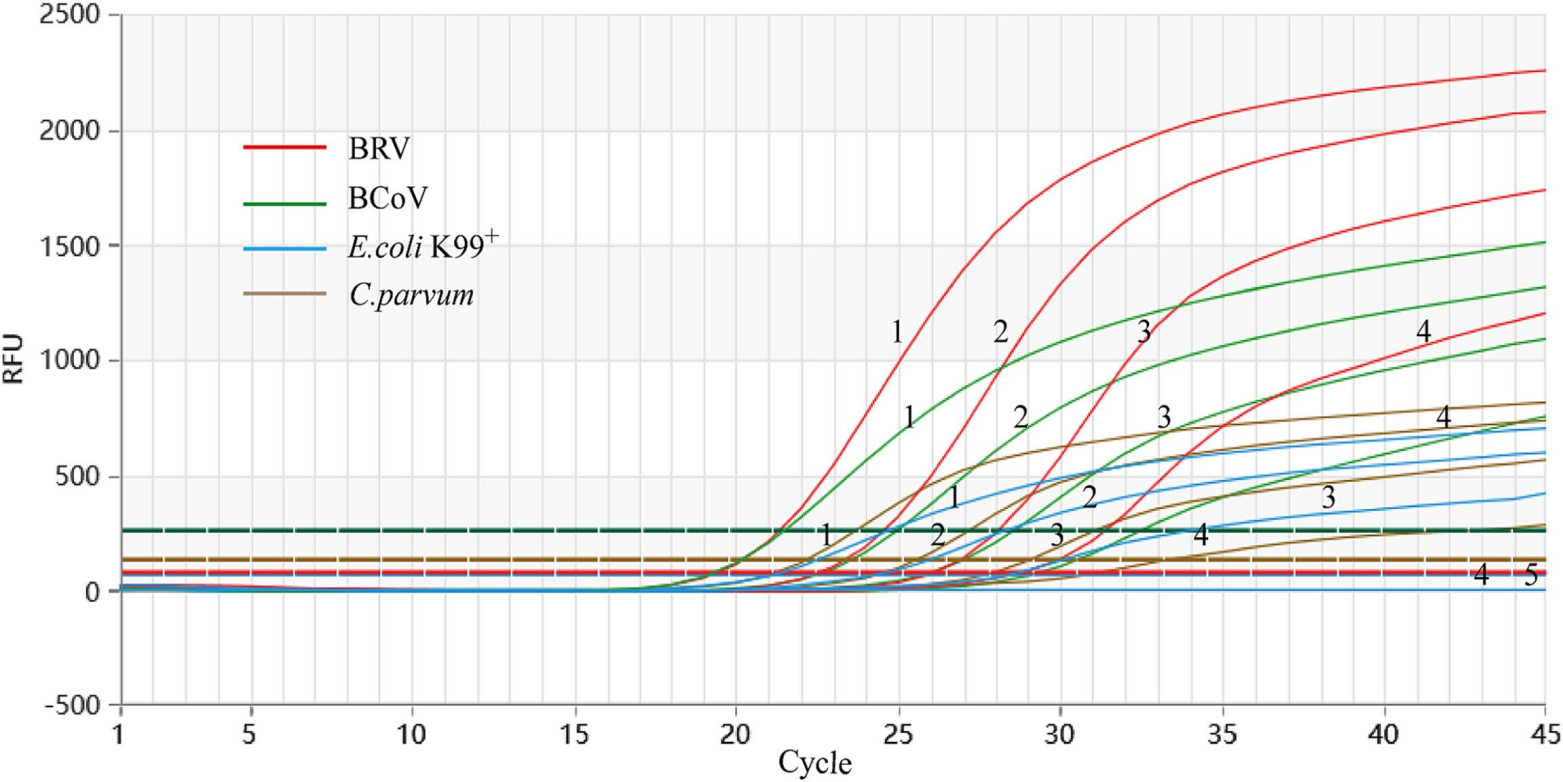
Sensitivity test of singleplex TaqMan Real-Time PCR Assay. X-axis: number of cycles; Y-axis: RFU; 1–4: fluorescence quantitative PCR amplification curve of each standard positive recombinant plasmid from 10^4^ to 10^1^ copies/μL of plasmid content; 5: ddH_2_O.

Using a gradient dilution of standard plasmids ranging from 10^4^ to 10^1^ copies/μL as templates, with ddH_2_O as a negative control, both singleplex and multiplex *Taq*Man fluorescent quantitative PCR amplifications were performed. The detection limits of the established method for BRV, BCoV, *E. coli* K99^+^, and *C. parvum* were determined to be 5.8 × 10^1^, 2.3 × 10^1^, 4.5 × 10^2^, and 2.6 × 10^1^ copies/μL, respectively (Figure 3). The established multiplex *Taq*Man quantitative PCR detection method still shows good amplification curves at this plasmids concentration (Figure 4).

### 3.6 Reproducibility analysis

The highest variation coefficient Cv value of the quadruple qPCR detection group was 1.08%, and the highest variation coefficient Cv value of the intergroup was 1.2%, indicating that the quadruple *Taq*Man fluorescence quantitative PCR detection method had good detection repeatability and high accuracy (Table 2).

**Table 2:** Repeatable experiments between batches and within batches.

Target	Templates (copies/μL)	Repetitions	Intra-assay		Interassay			
			x̄	s	Cv (%)	x̄	s	Cv (%)
BRV	10^4^	3	28.68	0.26	0.91	28.87	0.25	0.87
	10^3^	3	31.91	0.18	0.57	31.72	0.34	1.08
	10^2^	3	34.37	0.04	0.13	34.54	0.06	0.17
BCoV	10^4^	3	25.15	0.11	0.42	25.11	0.19	0.74
	10^3^	3	28.99	0.24	0.81	28.66	0.08	0.26
	10^2^	3	32.15	0.17	0.52	32.23	0.95	0.30
*E. coli* K99^+^	10^4^	3	26.94	0.09	0.35	28.67	0.34	1.20
	10^3^	3	30.68	0.33	1.08	30.56	0.24	0.77
	10^2^	3	33.84	0.28	0.84	33.80	0.19	0.56
*C. parvum*	10^4^	3	27.89	0.16	0.58	27.95	0.88	0.31
	10^3^	3	31.98	0.32	1.03	31.84	0.28	0.88
	10^2^	3	34.57	0.08	0.22	34.38	0.21	0.60

### 3.7 Validation of diagnostic specificity and sensitivity

**Figure 5:**
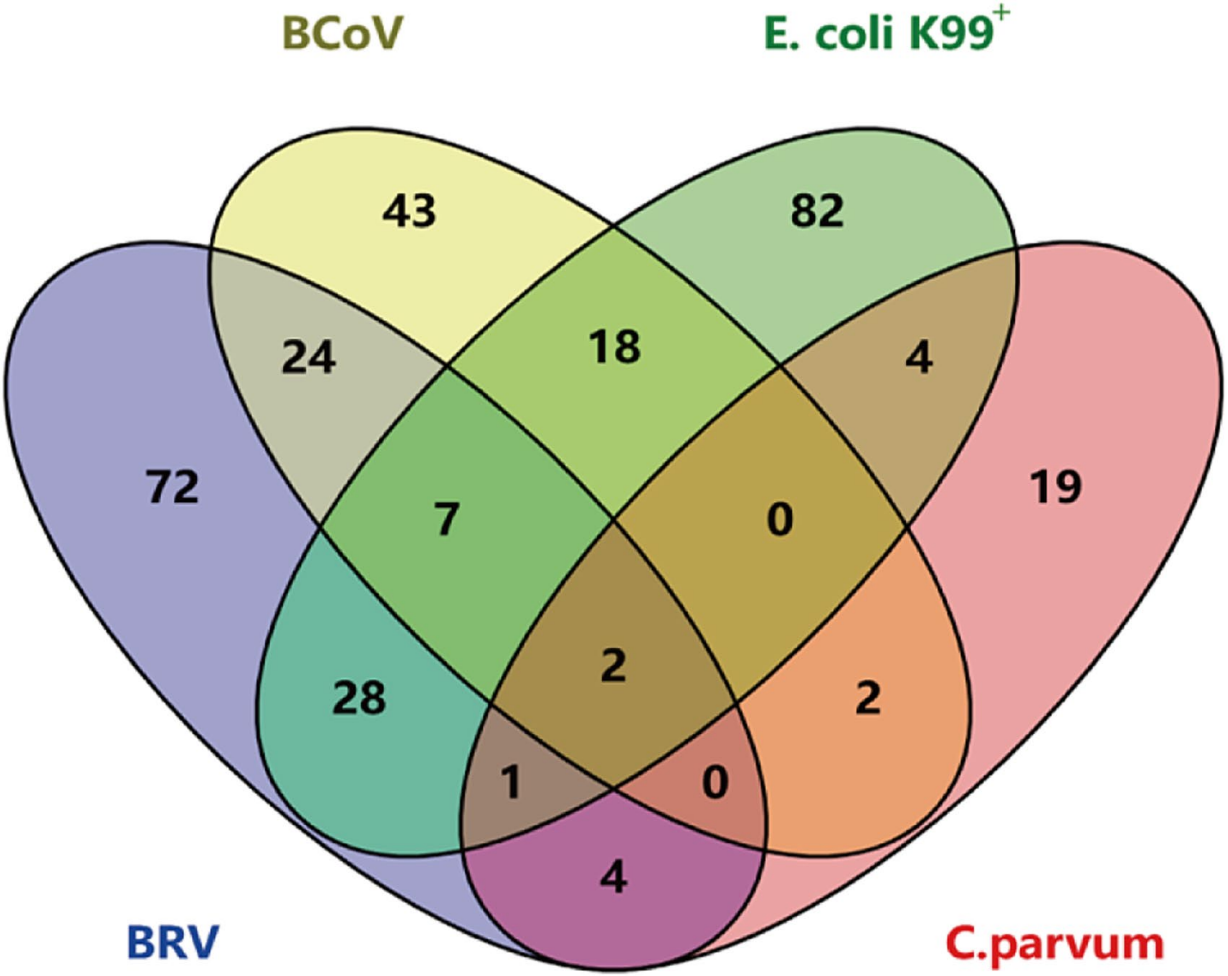
Results of multiple TaqMan fluorescence quantitative PCR for 400 clinical samples. The diagram illustrates the distribution of samples infected with individual pathogens and co-infected samples. Overlapping regions represent samples infected with multiple pathogens simultaneously.

The qPCR method established in this study and the commercial qPCR kit method were used to analyze 400 clinical samples collected from intensive farms in Shandong Province. The results of quadruple fluorescence quantitative PCR revealed that 72 of the 400 samples were BRV positive, with a positive detection rate of 18%; 43 were BCoV positive, with a positive detection rate of 10.75%; 82 were *E. coli* K99*^+^* positive, with a positive detection rate of 20.5%; and 19 were *C. parvum* positive, with a detection rate of 4.75%. The detection rates of BRV + BCoV, BRV + *E. coli* K99^+^, BRV + *C. parvum*, BCoV+*E. coli* K99^+^, BCoV+*C. parvum*, and *E. coli* K99^+^+*C. parvum* were 6, 7, 1, 4.5, 0.5, and 1%, respectively. The detection rates of BRV + BCoV+*E. coli* K99^+^, BRV + BCoV+*C. parvum*, BRV + *E. coli* K99^+^+*C. parvum*, and BCoV+*E. coli* K99^+^+*C. parvum* were 1.75, 0, 0.25, and 0%, respectively. The detection rate of BRV + BCoV+*E. coli* K99^+^+*C. parvum* was 0.5% (Figure 5).

## 4 Discussion

Diarrhea is one of the most serious diseases of newborn calves, causing significant economic losses due to mortality, poor growth, and treatment costs (33). During diarrhea outbreaks in herds, it is important to identify the infectious cause so that further appropriate control measures can be targeted (34). Real-time PCR is easily adapted to detect nucleic acid targets specific to each given pathogen, with high specificity and sensitivity; making it a vital tool for infection identification and detection (35). Therefore, we developed a multiple *Taq*Man qPCR protocol to simultaneously detect single or mixed infections of the four main pathogens that cause diarrhea in calves. The nucleic acid extraction procedure can simultaneously extract DNA and RNA from viruses, bacteria, and protozoa, saving time and cost. In this study, primers and probes were designed for the BRV VP6 gene, BCoV N gene, *E. coli* K99^+^ K99 gene and *C. parvum* 18S rRNA gene. After the selection of probes and primers and the optimization of the concentration ratio, a fluorescence quantitative PCR detection method based on a *Taq*Man probe was successfully constructed. Standard plasmids showed a good linear relationship in the range of 10^8^ ~ 10^2^copies/μL, with R^2^ values greater than 0.997. The method has good specificity and does not exhibit cross-reactions with other pathogens. The experimental results are stable within and between groups, and the coefficient of variation is less than 1.2%, indicating that the method has good repeatability. The methods used to detect *E. coli* K99^+^ and *C. parvum* are mostly common PCR (36, 37). Wang et al. established a triple qPCR method for BCoV, BRV and *E. coli* K99^+^, with a minimum detection limit of 10 copies/μL (27). Compared with Wang’s method, this method did not achieve higher sensitivity, likely due to the inherent limitations of multiplex fluorescence quantification. Establishing quadruple qPCR is more challenging. In this study, we screened various primer-probe combinations. Since the four signals have different wavelengths, their fluorescence signals can be detected simultaneously in the same reaction tube. High-specificity multiplex real-time PCR requires optimizing the concentration ratios of primers and probes to prevent mutual interference. Pedroso et al. established a fivefold PCR detection method for BRV, BCoV, *E. coli* K99^+^, *Pasteurella* and *Cryptosporidium* (26). In contrast, the introduction of the *Taq*Man probe in this method enhances the specificity of the target gene. This method relies on the collection of fluorescence signals and can determine the presence or absence of pathogenic infection on the basis of Ct values. The experimental steps are simple, do not easily contaminate the environment, and can effectively shorten the detection time (28). Demirci et al. established conventional molecular kits for rotavirus Group A, *E. coli* K99^+^, and *C. parvum* species based on LAMP method, which can be used under field conditions without a laboratory environment and provides results in a short time. However, compared with the PCR method, this method was less sensitive, which can result in false-positive or false-negative results (38).

In this study, 400 clinical diarrhea samples from Shandong Province were used to evaluate the performance of the developed multiplex qPCR method. Compared to commercial single pathogen-detection kits, the method showed diagnostic sensitivity and specificity greater than 90%, with a kappa value exceeding 0.9. Despite some false negatives and false positives, these results indicate a high level of agreement between the two methods (Table 3). The occurrence of false-positive and false-negative samples may be attributed to various factors. False positives could arise from sample or reagent contamination, non-specific amplification, or cross-reactivity of primers/probes with non-target sequences. Conversely, false negatives might be caused by inhibitors present in the sample, such as heme or polysaccharides in feces, or by incomplete nucleic acid extraction. We did not perform further tests to investigate the false positive and false negative samples, and a key limitation of this study is the lack of internal controls, which could have improved the reliability of the results. In this study, we used the established method to test 400 diarrhoeal fecal samples from Shandong. The detection rates for BRV, BCoV, *E. coli* K99^+^, and *C. parvum* were 18% (72/400), 10.75% (43/400), 20.5% (82/400), and 4.75% (19/400), respectively. Among these pathogens, *E. coli* K99^+^ had the highest detection rate. These results demonstrate that the four pathogens play a significant role in the occurrence of calf diarrhea.

**Table 3:** Method and evaluation results of various indicators of commercial reagent kits.

Assay	Pathogen	Results	Commercial kit			Performance characteristic		Kappa
			P	N	Total	DSe	DSp	
Multiple RT–qPCR	BRV	P	72	2	74	98.6	99.4	0.977
		N	1	325	326			
	BCoV	P	43	3	46	99.6	99.2	0.982
		N	2	352	354			
	*E. coli* K99^+^	P	82	2	84	96.4	99.4	0.963
		N	3	313	316			
	*C. parvum*	P	19	1	20	95	99.7	0.947
		N	1	379	380			
	BRV + BCoV	P	24	1	25	100	99.7	0.983
		N	0	375	375			
	BRV + *E. coli* K99^+^	P	28	0	28	100	100	1
		N	0	372	372			
	BRV + *C. parvum*	P	4	0	4	100	100	1
		N	0	396	396			
	BCoV+*E. coli* K99^+^	P	18	0	18	94.7	100	0.970
		N	1	380	381			
	BCoV+*C. parvum*	P	2	0	2	100	100	1
		N	0	398	398			
	*E. coli* K99^+^+*C. parvum*	P	4	0	4	100	100	1
		N	0	396	396			
	BRV + BCoV+*E. coli* K99^+^	P	7	0	7	100	100	1
		N	0	393	393			
	BRV + *E. coli* K99^+^+*C. parvum*	P	1	0	1	100	100	1
		N	0	399	399			
	BRV + BCoV+*E. coli* K99^+^+*C. parvum*	P	2	0	2	100	100	1
		N	0	398	398			

One limitation of this study is the lack of cross-reactivity testing to assess potential interactions between the primers, probes and non-target organisms. Although in analysis indicated high specificity, experimental validation would strengthen the evidence. This limitation may impact result interpretation, especially in cases of co-infections or closely related organisms. Future studies will include comprehensive cross-reactivity testing to further validate the assay’s specificity and reliability.

**Figure 6:**
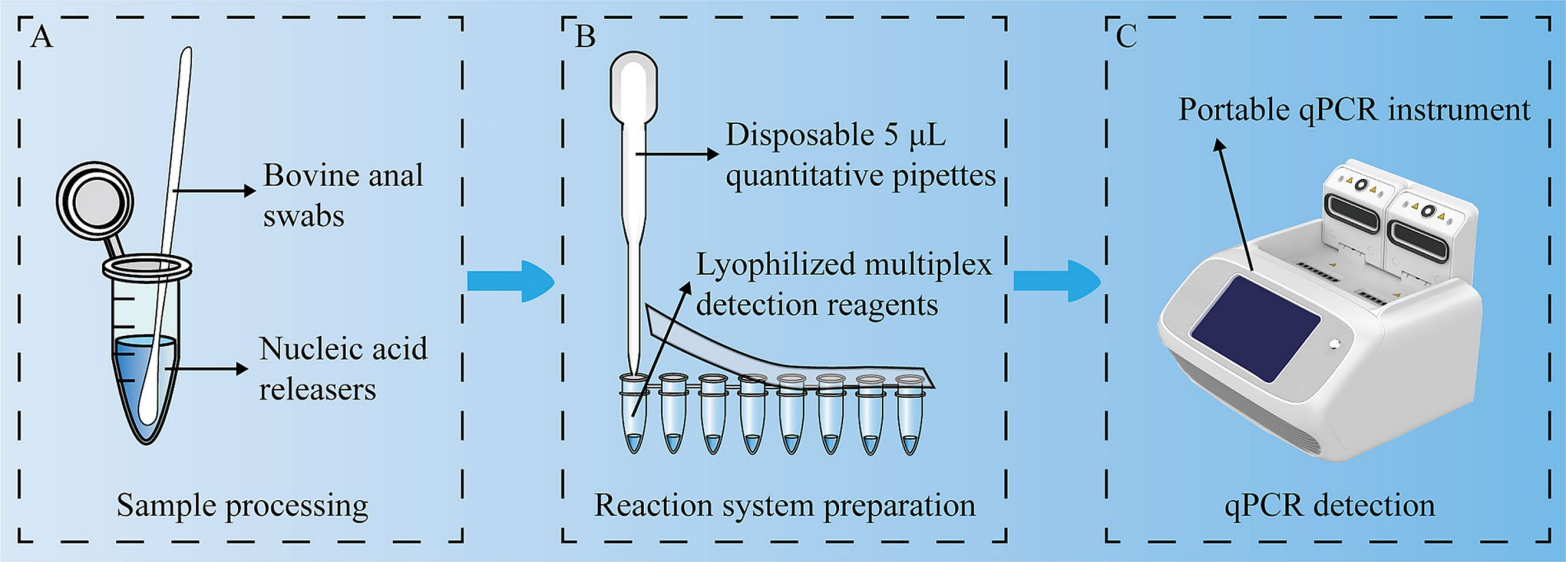
Experimental flow chart. A multiplex, one-step RT–qPCR assay was used to detect BRV, BCoV, *E. coli* K99^+^ and *C. parvum* simultaneously. **(A)** Sample processing; **(B)** Reaction system preparation; **(C)** qPCR detection.

In future studies, we propose a new farm detection scheme that includes a qPCR device and a set of reagent kits. The reagent kit contains lysis buffer, primers and probes for BRV, BCoV, *E. coli* K99^+^ and *C. parvum*, reverse transcriptase, and sampling swabs. This scheme does not require a centrifuge, professional operation, or complex nucleic acid extraction steps. The swabs are used to collect samples and then the swabs are placed in lysis buffer for 3 min. A pipette is used to transfer 5 μL of the sample to the reaction tube, and then qPCR detection is performed. The results can be obtained in approximately 1 h (Figure 6). This method eliminates many complex steps, reducing manual operations in sample processing and pipetting. In scenarios requiring simultaneous detection of multiple pathogens, quadruplex qPCR can improve detection efficiency. In the future, it has the potential to achieve automation and high-throughput detection.

## 5 Conclusion

In conclusion, this study successfully established a four-way detection method for BRV, BCoV, *E. coli* K99^+^ and *C. parvum*. This method has good specificity, high sensitivity and good repeatability and can quickly detect infections and mixed infections caused by bovine diarrhea pathogens via laboratory detection, providing convenience for the timely clinical treatment of bovine diarrhea. Consequently, the adverse effect of diarrhoeal pathogens on calves can be reduced.

## Data Availability

The original contributions presented in the study are included in the article/supplementary material, further inquiries can be directed to the corresponding author.
